# Can contagious itch be affected by positive and negative suggestions?

**DOI:** 10.1111/exd.14663

**Published:** 2022-09-01

**Authors:** Stefanie H. Meeuwis, Aleksandrina Skvortsova, Antoinette I. M. van Laarhoven, Henning Holle, Andrea W. M. Evers

**Affiliations:** ^1^ Health, Medical and Neuropsychology Unit, Institute of Psychology Leiden University the Netherlands; ^2^ Pain Research Group, Institute of Psychology Jagiellonian University Kraków Poland; ^3^ Department of Psychology McGill University Montreal Quebec Canada; ^4^ Leiden Institute for Brain and Cognition Leiden University Medical Center Leiden the Netherlands; ^5^ Department of Psychology University of Hull Hull UK; ^6^ Medical Delta Healthy Society Leiden University, Technical University Delft & Erasmus University Rotterdam Rotterdam the Netherlands

**Keywords:** contagious itch, expectancy, itch, nocebo effects, placebo effects

## Abstract

Contagious itch can be evoked by observing people scratching. Verbal suggestions about to‐be‐received itch can influence itch intensity, as shown by placebo research, but it is unknown whether this extends to contagious itch. The current study aimed to replicate prior findings that listening to scratching and rubbing sounds elicits contagious itch, and to investigate whether suggestions can modulate this process. Healthy participants (*n* = 140) received positive or negative suggestions about itch in response to the sounds (aimed to decrease or increase expected itch, respectively), or no specific suggestions as a control. Participants listened to a number of audio fragments with scratching and rubbing sounds. The amount of expected itch as well as itch sensation after each audio fragment were measured by self‐report. Suggestions had no effect on the expected itch. Both rubbing and scratching sounds significantly elicited itch in all groups. Scratching sounds induced more itch than rubbing sounds exclusively in the control group. These findings indicate that short suggestions might be not effective enough to modify the expectations of people regarding contagious itch. Furthermore, suggestions modulate contagious itch to some degree, but not in the hypothesized direction. Potential similarities and differences in the neurobiological mechanisms of contagious itch and nocebo effects are discussed.

## 1 INTRODUCTION

Contagious itch is a phenomenon in which non‐physical cues (i.e., pruritogen‐free or non‐cutaneous stimuli), such as observing or hearing someone scratching their skin, can cause itch. It has repeatedly been demonstrated that it is possible to evoke contagious itch by presenting people with videos of someone scratching or pictures of itch‐related stimuli and situations,^1^, ^2^, ^3^, ^4^ or by having people listen to sounds of scratching or rubbing.^5^ Understanding contagious itch is important for several reasons. Itch is a common symptom of many dermatological conditions, such as psoriasis, dermatitis or allergies, and other conditions such as infections and endocrine disorders.^6^ Chronic itch in particular is associated with considerable suffering and decreased quality of life.^7^, ^8^ Because contagious itch seems to be amplified in patients with chronic itch,^2^ it is important to find ways to alleviate it.

Psychological processes play an important role in how itch is experienced. A biopsychosocial model of itch has been proposed that emphasizes the role of psychological factors in itch experience.^8^, ^9^ For instance, stress and anxiety exacerbate chronic itch in patients with dermatological conditions.^10^, ^11^ The influence of individuals' expectancies on itch is also underlined by research into placebo and nocebo effects.^12^, ^13^, ^14^, ^15^, ^16^, ^17^ Placebo effects are positive effects (e.g., reduced itch) that do not emerge due to active treatment components, but are rather elicited by non‐active components of the treatment or its psychosocial context, for instance when these factors evoke positive expectancies about treatment outcomes.^18^ Nocebo effects are negative effects such as increased itch, side effects or reduced treatment efficacy elicited by the context.^19^ Placebo and nocebo effects have often been induced in cutaneous itch through expectancy modulation, that is, by giving positive or negative suggestions about some form of a sham treatment.^12^, ^13^, ^15^ For example, Darragh and colleagues^15^ found that participants experienced less itch after a cream was applied, when they were told that it was an antihistamine.

Little is known about whether itch evoked by non‐cutaneous stimuli may also be sensitive to expectancy effects. So far, a single study investigated whether suggestions can influence contagious itch in a 3 (catastrophizing information, simple information, no information) by 2 (patients, healthy controls) design.^20^ The authors found that catastrophizing information (i.e., suggestions that the induced itch would be extremely unpleasant) led to increases in scratching during and after itch‐related audiovisual stimuli, compared with a group that was simply informed that they may experience itch in response to the stimuli. The group that was not warned about itch did not differ from the group that received catastrophizing information, which could be related to heightened arousal.^20^ Moreover, the effects of suggestions were only seen in patients with dermatological conditions, and not in healthy controls, suggesting that patients may be more sensitive to itch‐related catastrophizing information.^20^ Itch intensity was not altered in response to the catastrophizing information though, in either patients or healthy controls. While these findings provide us with information about the potential impact of negative information and expectations, it is not clear how contagious itch may change when positive information evokes expectancies of low itch. For instance, it is possible to prevent nocebo effects by explaining them as typical human responses to health warnings.^21^, ^22^, ^23^ Similarly, such positive explanations may help to alleviate contagious itch. Negative suggestions, on the contrary, could draw attention to and as such increase itch elicited by audiovisual cues.^24^

The present study had two main goals: first, we aimed to replicate the results of previous research and investigate whether sounds of scratching induce sensations of itch to a higher extent than rubbing sounds. Secondly, we studied whether it is possible to change the expectancies of participants regarding contagious itch and modulate contagious itch intensity using negative and positive suggestions. Additionally, we explored whether interindividual differences in, for instance, anxiety, worrying and optimism, may moderate the effects of suggestions on contagious itch.

## 2 MATERIALS AND METHODS

### 2.1 Study design

The study was approved by the Psychology Research Ethics Committee of Leiden University (number 2020‐05‐08‐A.W.M. Evers‐V3‐2410). The study protocol was preregistered on Open Science Framework (osf.io/muqcj). A randomized between‐within subject study design was applied. Participants were randomly allocated to one of three groups: (1) negative suggestions group, (2) positive suggestions group or (3) control group (no specific suggestions). The distribution of males and females (i.e., based on self‐reported sex) was equal across groups.

### 2.2 Participants

Healthy participants between 18 and 60 years old were recruited for this study. Participants were recruited online using social media sites such as Facebook, and Sona; a participant database of Leiden University (ul.sona‐systems.com). The exclusion criteria were as follows: chronic itch experienced within the past three months, use of painkillers, sleep‐inducing medication, alcohol or other drugs 24 h before participation, caffeine consumption 1 h before participation.

A power analysis was conducted in GPower version 3,^25^ with the aim to determine the optimal sample size to detect differences in the intensity of contagious itch among the three study groups. Input for the power analysis was derived from two studies: (1) a study that investigated the influence of positive suggestions on itch intensity following topical histamine application^14^ was used for calculating a sample size needed to detect the between‐group differences (the effect of suggestions); (2) a study that investigated the effect of scratching and rubbing sounds on the subjective itch intensity^5^ was used for calculating a sample size needed to detect any within‐group differences (i.e., any difference in contagious itch elicited by scratching relative to rubbing sounds), in order to ensure that we could replicate prior research findings. The power analysis indicated that to detect between‐group differences in subjective itch experience using analysis of variance with 3 groups, with an estimated effect size of *f* = 0.42,^14^ a critical alpha level of *α* = 0.05 and a power of 1−*β* = 0.95, a number of 31 participants per group would be needed. To detect within‐group differences in contagious itch using an analysis of variance with an estimated effect size of *f* = 0.17,^5^ a critical alpha level of *α* = 0.05 and a power of 1−*β* = 0.95, a number of 46 participants per group would be needed.

Taking into consideration that the manner of providing suggestions differs from prior work (i.e., a combination of verbal and written suggestions in Darragh and colleagues,^14^ versus completely online in the current study), and that the auditory itch induction in the previous study^5^ was also induced in a laboratory setting, the number of participants per group was adjusted to a more conservative 50 participants per group.

### 2.3 Experimental interventions

The suggestions and the quality of the sounds were checked in a pilot that included 17 participants. The length of the suggestions was matched in all groups except the control group, which received slightly shorter instructions. The full text of the suggestions and results of the pilot are presented in Appendix 1.

#### 2.3.1 Negative suggestions group

In this condition, participants were given information about contagious itch and were informed that most people experience itch after hearing the scratching and rubbing sounds, even when being aware of the phenomenon.

#### 2.3.2 Positive suggestions group

In this condition, participants received information on contagious itch, and were informed that being aware of the contagiousness of itch will minimize its impact.

#### 2.3.3 Control group

In this condition, participants did not receive any explicit suggestions and were merely told that in the following part they would be asked to listen to various sounds, and to rate the amount of itch they experienced afterwards.

### 2.4 Itch induction

The sound task used in this study for itch induction is described in detail in previous research.^5^ The sounds comprised recordings of scratching and rubbing different targets, including 3 body parts (beard, hand, leg) and 3 non‐body materials (polyester, denim, leather). Each sound was administered three times, with different compositions: high frequency (HF) tones above 1000 Hz were either increased or decreased in amplitude (i.e., volume) by 10 dB relative to other tones in the audio recordings. This resulted in 3 different versions of each sound: HF tones' amplitude −10 dB, HF tones' amplitude unchanged and HF tones' amplitude +10 dB. In total, participants listened to 36 sounds, of which 18 were scratching and 18 were rubbing sounds, and each sound lasted for 20 s. The sounds were presented to participants in three blocks, using a pseudorandom order (i.e., every block consisted of 6 scratching and 6 rubbing sounds, and the blocks were randomized).

### 2.5 Measurements and questionnaires

*Expected itch* of participants was measured after the suggestions were given, but before the sounds were presented. Participants were asked to predict how much itch they think they will experience during listening to the sounds on a 0–10 numeric rating sale (NRS; 0—“no itch” to 10—“worst itch imaginable”).

*Experienced itch* was measured after the presentation of each sound. Participants were asked to indicate on a 0–10 NRS how much itch they experienced while listening to the sound (0—“no itch” to 10—“worst itch imaginable”).

#### 2.5.1 Questionnaires

*Itch experience questionnaire* was developed by one of the authors (HH) for familiarizing participants with an 11‐point NRS for itch. Participants were asked whether they ever experienced: (1) a mosquito bite; (2) an itchy scalp (for example, after using wrong kind of shampoo); (3) itchy feet/toes (for example, athletes' foot). In case participants positively replied to these questions, they were asked to use the NRS and rate how much itch these experiences elicited (0—“no itch” to 10—“worst itch imaginable”). As this scale was primarily used for familiarizing participants with rating itch, no total score was computed.

*Interindividual differences* in the following constructs were assessed: neuroticism and extraversion (*short version of the Eysenck Personality Questionnaire, EPQ‐RSS* ^26^), optimism (*Revised Life Orientation Test, LOT‐R* ^27^), worrying (*Penn State Worry Questionnaire, PSWQ* ^28^), state anxiety (*State Trait Anxiety Inventory short version, STAI‐Ss* ^29^), and the severity of skin sensitivity and skin irritation over the past 3 days (*Sensitive Scale‐10, SS‐10* ^30^).

### 2.6 Procedure

The study was advertised online as a study that investigated individual differences in itch sensitivity. Interested volunteers were sent a link to the online survey platform Qualtrics (Provo, UT & Seattle, WA, USA), containing the study's information letter. In case they wanted to participate, they were asked to sign an online informed consent form.

Participants were then asked to fill out a short online screening questionnaire, that checked the inclusion and exclusion criteria. In case of ineligibility for participation, they were automatically referred to the end of the survey and participation discontinued. Eligible participants were asked to listen to a sample sound (i.e., a beeping tone) and to report how well they could hear it. They were asked to wear headphones (noise‐cancelling if available) during the task to improve the quality of the sounds. Then, they were given the itch experience questionnaire. Subsequently, participants were randomly allocated to one of the three groups: negative suggestions, positive suggestions or a control group and received various instructions depending on their group allocation (see paragraph “Experimental interventions”). Participants then rated how much itch they expected to experience while listening to the audio recordings. Next, participants listened to the sounds of skin and material scratching and rubbing. After each of the 20 s sounds, participants were asked to report the level of itch that they felt on the NRS. Subsequently, the participants filled out the rest of the questionnaires. Following this, participants were debriefed. The study lasted around half an hour and was conducted in English. Participants were compensated with either 1 course credit (for Psychology students of Leiden University) or the option to take part in a lottery and win 15 euros.

### 2.7 Statistical analysis

All analyses were conducted using SPSS 26.0 for windows (IBM SPSS Inc., Chicago, Illinois, USA). Prior to analyses, all variables were checked for normal distribution and the assumptions of the preplanned statistical methods were checked. All variables were normally distributed except for age and mean experienced itch scores. *χ* ^2^ tests, analysis of variance (ANOVA) and a Kruskal–Wallis non‐parametric test (i.e., for age) were used to examine between‐group differences in demographic variables and individual characteristics.

Because mean itch scores were not normally distributed, and no non‐parametric equivalent of the mixed model repeated‐measures ANOVA exists, square root transformation was performed on itch scores. First, a one‐sample *t*‐test was used to compare the whole‐sample mean for expected itch to zero, to check whether participants expected the sounds to elicit itch. This was followed by an ANOVA to assess whether the positive suggestions, negative suggestions and control group differed in expected itch.

Second, we assessed whether contagious itch could be elicited with a one‐sample *t*‐test, in which we compared whole‐sample mean itch for scratching and rubbing sounds across all groups to zero. Next, a 2 (sound type: scratching vs. rubbing) × 3 (HF amplitude: −10 dB, original, +10 dB) within‐subjects repeated‐measures ANOVA was conducted to assess whether prior study findings^5^ could be replicated. Because the suggestions in the experimental groups were aimed at manipulating itch levels, this analysis was conducted within the control group exclusively (a secondary analysis of the replicability of prior research findings including the positive and negative suggestion groups was conducted. The results of this analysis can be found in Appendix 2). Bonferroni post hoc tests were performed for each factor within this analysis.

Third, the effects of verbal suggestions on itch were assessed using a 3 × 2 mixed ANOVA, with group allocation (positive suggestions, negative suggestions, control) as between‐subjects factor and sound type (scratching, rubbing) as within‐subjects factor. Itch ratings were averaged across HF amplitude for this analysis. Planned Bonferroni‐corrected comparisons for itch were performed: (1) between positive suggestions and the control group, averaged across sound type; (2) between negative suggestions and the control group, averaged across sound type; (3) between positive suggestions and the control group for each sound type (scratching, rubbing) separately; (4) between negative suggestions and the control group for each sound type (scratching, rubbing) separately; and (5) between scratching and rubbing sounds within each separate group. To estimate whether the care with which participants read the instructions may influence the results, a sensitivity analysis was performed based on the total duration of the survey. Participants who were overly quick or slow (>1.5 standard deviation (SD) slower or faster than the mean duration time) were excluded in this sensitivity analysis.

Fourth, explorative analyses were conducted to investigate whether individual characteristics modulated the effects of suggestions on itch elicited by the scratching and rubbing sounds. For these analyses, itch ratings were also averaged across HF amplitude. As a first step, Pearson's correlation coefficients were calculated to explore whether interindividual differences were associated with average itch elicited by scratching and rubbing sounds in general. To explore whether interindividual differences influenced the effects of the suggestions on contagious itch elicited by the scratching and rubbing sounds, multiplicative moderation analysis was then conducted with the MEMORE macro.^31^ The moderation analyses were conducted twice: once to compare the positive suggestions group and once to compare the negative suggestions group with the control group. Prior to the analysis, assumptions for ordinary least square (OLS) regression were checked. Moderation effects were probed using the “pick‐a‐point” approach and set at −1 SD, mean and +1 SD for the continuous variables. Confidence intervals (CI's) were generated by percentile bootstrapping set at 5000 samples.

All values are reported as arithmetic means ± SD unless stated otherwise. An alpha <0.05 is considered statistically significant. Because the itch scores were negatively skewed and violated the assumption of normal distribution of the residuals, square root transformations were performed on the data prior to all analyses.

## 3 RESULTS

### 3.1 Participants

In total, 157 participants started the study and were randomized into the three groups. Four participants did not complete the study, and data of 17 participants were excluded post hoc because they did not meet the inclusion criteria. The final sample consisted of 140 participants (84.3% female) with an age between 18 and 57 years old (20.64 ± 5.02). Most of the participants had university education (80.9%) and were of Dutch nationality (59.6%). *χ* ^2^ tests, analysis of variance and Kruskal–Wallis non‐parametric tests demonstrated no differences between the groups in demographics or on the assessed questionnaires (all *p* ≥ 0.12; Table 1).

**TABLE 1:** Demographics and interindividual differences in each group

	Positive suggestions group (*n* = 52)	Negative suggestions group (*n* = 46)	Control group (*n* = 42)	Group difference (*p‐*value)
Age	21.12 ± 5.95	20.53 ± 5.19	20.17 ± 3.36	0.47
Sex (female)	43 (82.70)	40 (87.00)	35 (83.30)	0.83
Education				0.12
Secondary education	3 (5.90)	5 (11.40)	6 (14.60)	
Higher education	3 (5.90)	7 (15.90)	1 (2.40)	
University	45 (88.20)	32 (72.70)	33 (80.50)	
Other	0 (0.00)	0 (0.00)	1 (2.40)	
Nationality^a^				0.73
Dutch	28 (54.90)	29 (65.90)	24 (58.50)	
German	5 (9.80)	6 (13.60)	5 (12.20)	
Dual citizenship	2 (3.90)	2 (4.50)	3 (7.30)	
Other	16 (31.40)	7 (16.00)	9 (22.00)	
Itch experience^b^				
Mosquito bite (yes)	48 (92.30)	43 (93.50)	42 (100.00)	0.20
Mean ± SD	5.50 ± 1.79	6.14 ± 1.58	5.90 ± 1.71	0.19
Itchy scalp (yes)	37 (71.20)	35 (76.10)	31 (73.80)	0.86
Mean ± SD	4.11 ± 2.01	5.00 ± 1.70	4.84 ± 1.79	0.10
Itchy feet/toes (yes)	22 (43.10)	27 (58.70)	22 (52.40)	0.30
Mean ± SD	5.05 ± 1.53	5.15 ± 1.70	4.64 ± 1.92	0.56
Interindividual differences				
Neuroticism	5.92 ± 3.40	6.16 ± 3.31	5.71 ± 3.52	0.83
Extraversion	7.78 ± 4.04	7.80 ± 3.43	7.90 ± 3.51	0.99
Optimism	14.29 ± 4.47	14.50 ± 4.16	13.73 ± 4.12	0.69
Worrying	52.51 ± 13.67	55.52 ± 13.50	52.20 ± 12.40	0.43
State anxiety	12.00 ± 3.82	12.09 ± 3.33	10.85 ± 3.25	0.19
Sensitive skin rating	14.28 ± 11.23	18.64 ± 15.29	14.57 ± 10.86	0.19

*Note*: Values are listed as mean ± standard deviation or as *n* (%). *n* = 4 missing on all variables. % are adjusted for missing data. Group differences were assessed by *χ* ^2^ tests for categorical and analysis of variance for continuous variables. For age, a Kruskal–Wallis test was performed because of non‐normal distribution of the data.

^a^ The largest categories have been described ‐all other nationalities (*n* = 20 in total, with *n* < 5 for each respective nationality) have been listed as “other.”

^b^ When participants answered “yes” on the question whether they experienced itch before, for example, from a mosquito bite or itchy scalp (the proportion that answered “yes” is presented in the table), they were then asked to rate itch intensity on a 0–10 numeric rating scale (Mean ± SD in the table).

### 3.2 Expected itch

A one‐sample *t*‐test showed that, prior to listening to the scratching and rubbing sounds, participants in all groups expected to experience itch when listening to the sounds; *t*(135) = 20.03, *p* < 0.001. The suggestions did not modulate the amount of itch participants expected to experience prior to listening to the sounds *F*(2,133) = 2.09, *p* = 0.13, *η* ^2^_partial_ = 0.03. For an overview of the means per group for each outcome, see Table 2.

**TABLE 2:** Means ± standard deviations for the study outcomes per group

	Positive suggestions group (*n* = 52)	Negative suggestions group (*n* = 46)	Control group (*n* = 42)
*Process measure* ^a^			
Postsuggestions expected itch	3.47 ± 2.12	4.05 ± 2.33	4.43 ± 2.42
*Itch scores for scratching sounds* ^b^			
Averaged across HF amplitude	2.35 ± 2.05	2.16 ± 1.84	2.52 ± 2.41
Averaged across HF amplitude (sqrt)	1.76 ± 0.53	1.71 ± 0.49	1.78 ± 0.62
Separate for HF amplitude			
… −10 dB	2.35 ± 2.00	2.08 ± 1.68	2.35 ± 2.34
… Original	2.33 ± 2.17	2.17 ± 1.82	2.50 ± 2.47
… +10 dB	2.38 ± 2.12	2.22 ± 2.09	2.73 ± 2.56
… −10 dB (sqrt)	1.39 ± 0.66	1.30 ± 0.63	1.31 ± 0.81
… Original (sqrt)	1.37 ± 0.69	1.33 ± 0.64	1.37 ± 0.79
… +10 dB (sqrt)	1.38 ± 0.70	1.31 ± 0.71	1.43 ± 0.83
*Itch scores for rubbing sounds* ^b^			
Averaged across HF amplitude	2.22 ± 1.91	2.06 ± 1.76	2.00 ± 2.10
Averaged across HF amplitude (sqrt)	1.72 ± 0.50	1.68 ± 0.48	1.64 ± 0.56
Separate for HF amplitude			
… −10 dB	2.03 ± 1.78	1.95 ± 1.64	1.73 ± 2.01
… Original	2.24 ± 2.03	2.02 ± 1.72	1.97 ± 2.08
… +10 dB	2.37 ± 2.06	2.17 ± 1.98	2.29 ± 2.30
… −10 dB (sqrt)	1.28 ± 0.63	1.24 ± 0.64	1.08 ± 0.77
… Original (sqrt)	1.34 ± 0.68	1.27 ± 0.65	1.18 ± 0.77
… +10 dB (sqrt)	1.40 ± 0.65	1.29 ± 0.72	1.30 ± 0.78

Abbreviations: dB, decibel; HF, high frequency; sqrt, square root transformed.

^a^ *n* = 4 missing.

^b^ *n* = 6 missing.

### 3.3 Experienced itch

An overview of the itch scores can be found in Table 2. The one‐sample *t*‐tests showed that on average, significant itch was elicited across all groups by the scratching sounds [*t*(133) = 37.35, *p* < 0.001] and rubbing sounds [*t*(133) = 38.08, *p* < 0.001].

Within the control group, a 2 × 3 mixed ANOVA demonstrated a significant main effect of sound type on itch (square root transformed, i.e., itch_sqrt_) [*F*(1,40) = 15.19, *p* < 0.001, *η* ^2^_partial_ = 0.28]: the scratching sounds elicited more itch (2.52 ± 2.41) compared with the rubbing sounds (2.00 ± 2.10). Moreover, a main effect of HF amplitude was found [*F*(2,40) = 10.07, *p* < 0.001, *η* ^2^_partial_ = 0.34]. Post hoc pairwise comparisons revealed that for every +10 dB increase in HF amplitude, itch increased as well (with mean difference in itch_sqrt_ ranging between 0.18 and 0.08; all *p* ≤ 0.014; see Table 1 for itch ratings). No interaction between sound type and HF amplitude was observed [*F*(1,39) = 2.29, *p* = 0.12, *η* ^2^_partial_ = 0.11].

### 3.4 Effects of verbal suggestions on contagious itch

The 3 × 2 mixed ANOVA demonstrated no significant main effect of group on itch_sqrt_; *F*(2,130) = 0.07, *p* = 0.94, *η* ^2^_partial_ < 0.01. This indicates that overall, the suggestions did not modulate the experience of contagious itch. The planned comparisons confirm that itch in general did not differ between the positive suggestions and control group (both *p* ≥ 0.11), nor between the negative suggestions and control group (both *p* ≥ 0.11).

**FIGURE 1:**
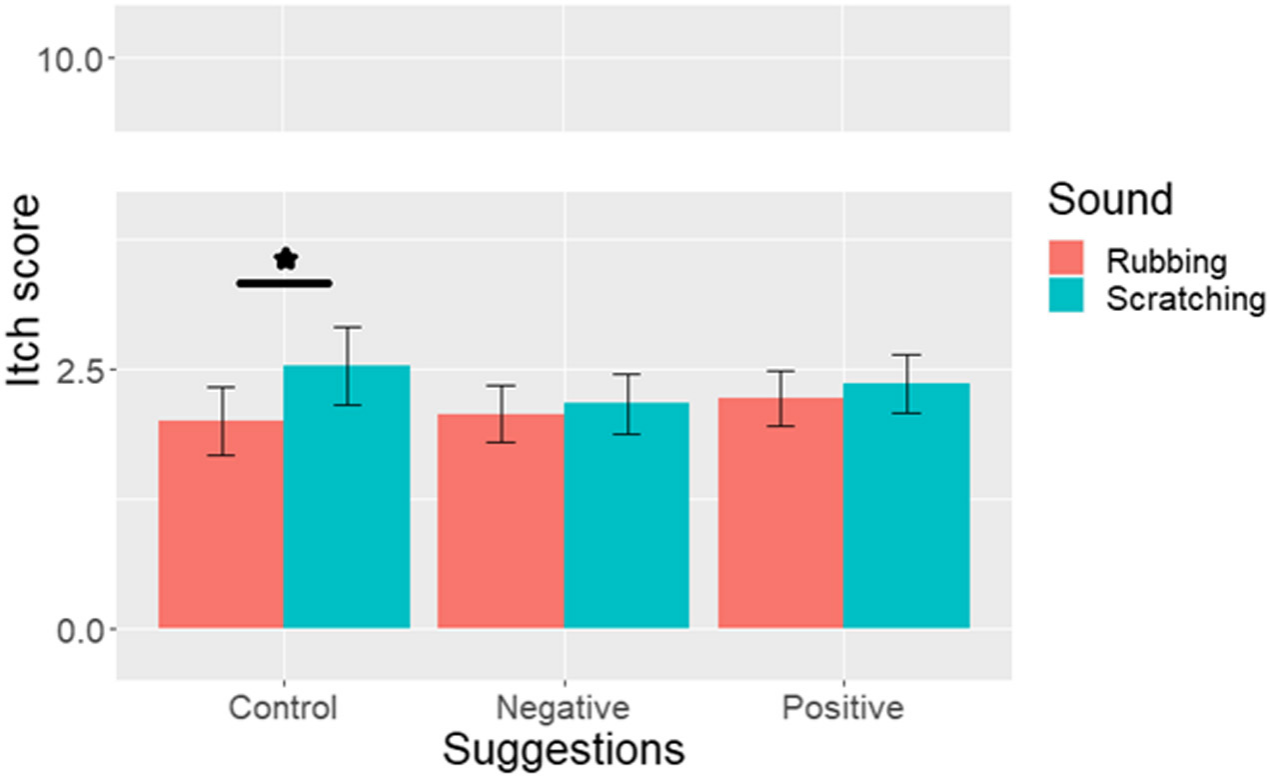
Mean experienced itch ± standard error in the positive suggestions (*n* = 51), negative suggestions (*n* = 41) and control (*n* = 41) groups, plotted across sound type (scratching vs. rubbing sounds). *p < 0.05

A main effect of sound type was found within the analysis; *F*(1,130) = 20.43, *p* < 0.001, *η* ^2^_partial_ = 0.14. Across all groups, participants reported more itch following the scratching sounds (2.35 ± 2.10) compared with the rubbing sounds (2.10 ± 1.91). A significant group × sound type interaction [*F*(2,130) = 5.62, *p* = 0.005, *η* ^2^_partial_ = 0.08] demonstrated that these findings can be attributed to differences between the sound types within the control group exclusively: the planned comparisons showed, that within this group, more itch was elicited by the scratching than the rubbing sounds (*p* < 0.001; Figure 1). In the positive and negative suggestion groups, on the contrary, itch ratings following scratching and rubbing sounds did not differ significantly (both *p* ≥ 0.19). No differences were detected between the positive suggestions and control group, or the negative suggestions and control group, in itch elicited by the scratching and rubbing sounds separately (all *p* ≥ 0.11; Figure 1). A sensitivity analysis revealed that these findings did not change, when participants who filled out the survey very quickly or slowly (>1.5 SD of the mean duration; 33.2 ± 14.65 in min) were excluded.

### 3.5 Moderation of group effects by interindividual differences

In general, interindividual differences (i.e., in anxiety, worrying, optimism and personality) were not correlated with itch (all *p* ≥ 0.067; Table S1). Moreover, interindividual differences did not moderate the effects of positive or negative suggestions on contagious itch (Tables S2 and S3), apart from sensitive skin. Sensitive skin ratings were positively correlated with itch following scratching (*r* = 0.22, *p* = 0.012) and rubbing (*r* = 0.19, *p* = 0.028) sounds: participants who rated their skin as more sensitive reported more itch. Moderation analysis revealed a significant interaction between group, sensitive skin ratings and sound type (for both positive and negative suggestions analyses: *p* < 0.001): participants in the control group rated itch elicited by scratching sounds significantly higher than itch elicited by the rubbing sounds, but only when they reported highly sensitive skin (+1 SD). When sensitive skin ratings were low (−1 SD), there was no difference between itch elicited by scratching or rubbing sounds. This effect was found only in the control group and was absent in the positive and negative suggestions groups (Appendix 3; Figures S3–S6).

## 4 DISCUSSION

The current study had a twofold aim: (1) to replicate the results of previous research that showed that listening to scratching and rubbing sounds can elicit sensations of itch, and (2) to investigate whether participants' expectancies about contagious itch can be changed and contagious itch intensity modulated using positive and negative suggestions. The results illustrate that listening to scratching and rubbing sounds causes contagious itch. Moreover, itch intensity depends on the sound type (scratching sounds elicited more itch than rubbing sounds) and on the amplitude of the high‐frequency tones in the recordings (when HF amplitude was higher, more itch was elicited, in particular for rubbing sounds). Contrary to our second hypothesis, positive and negative suggestions did not modulate participants' itch expectancies. The findings regarding itch were not straightforward: while itch ratings of participants in the control group displayed the same pattern as in previous research (i.e., increased itch in response to scratching versus rubbing sounds), the itch ratings following positive and negative suggestions did not.

That contagious itch can be induced by scratching and rubbing sounds is in line with previous research findings.^5^, ^32^ Similar to the study conducted by Swithenbank and colleagues,^5^ scratching sounds elicited itch to a higher extent than rubbing sounds and increases in HF amplitude were associated with higher auditory itch contagion, which confirmed our first hypothesis. Contrary to our second hypothesis, the positive and negative suggestions about contagious itch failed to modulate participants' itch expectancies as well as their general itch experience. These findings are somewhat unexpected in light of previous work that shows that verbal suggestions can modulate expectancies about, and the experience of, cutaneous itch e.g.,^12^, ^33^, ^34^ On the contrary, in previous research catastrophizing information only influenced scratching responses and did not affect contagious itch.^20^ These effects were moreover only found in patients with dermatological conditions, but not in healthy controls. Speculatively, patients may be more sensitive to catastrophizing information,^20^ and this may also be the case for information that is positively framed. However, positive expectancies about itch may be more easily induced in itch‐free populations; given that patients likely have a more extensive history with this symptom and may therefore hold more persistent negative expectancies towards itch‐evoking stimuli.

An interaction between suggestions and sound type (scratching versus rubbing) was found, which indicated that the pattern in itch ratings found in prior research^5^ and the control group—that is, that scratching sounds elicit significantly more itch compared with rubbing sounds—was absent following suggestions. Instead, itch ratings following rubbing sounds were somewhat higher, and itch ratings following scratching sounds somewhat lower after positive and negative suggestions relative to the control group (although between‐group comparisons were non‐significant). Thus, the suggestions equalized itch responses to scratching and rubbing sounds. Participants may have assumed following suggestions that all sounds would elicit itch, instead of monitoring carefully how each sound felt, which would account for this finding. However, this interpretation of the interaction effect is complicated by the lack of a baseline and it is unknown if there would be differences between the positive and negative suggestions groups in their itch sensitivity prior to receiving suggestions. In the majority of prior research, participants have had some experience with the itch sensations, either in the form of a baseline or control itch induction e.g.,^14^ or by experiencing these sensations in daily life prior to taking part in the study (e.g., as in case of the wind turbine syndrome^22^). In the current study on the contrary, it may have been difficult for participants to correctly estimate how much itch they would experience while listening to the audio recordings, because they have had no experience with listening to these sounds before. Future research on auditory induced contagious itch should let participants listen of the sounds, before the main manipulations are done, to establish a baseline. In addition, previous research took place in the laboratory, with the suggestions given either directly by an experimenter or presented in a written form. Our study, on the contrary, was conducted online, and participants were asked to read the verbal suggestions on their computers at home. Home settings may be more distractive, which could have further reduced the effectiveness of the suggestions. Although a sensitivity analysis revealed no significant change in findings when participants who finished the survey very quickly or slowly were excluded, and prior work indicates that presenting information online can modulate for instance the need for information about nocebo effects,^35^ some degree of inattention to the suggestions is still possible. Finally, it has been proposed that attention and expectancy may be separate constructs, that are both able to influence contagious itch.^24^ While the suggestions aimed to elicit positive expectancies and lower itch, they may have also redirected more attention towards physical sensations, which has been shown to be associated with increased itch sensitivity.^36^ Effects of positive expectancies could then have been negated. Interference by redirection of attention would not explain the direction of effects in the negative suggestions group; however, since there an additive effect of attention and negative expectancies on itch would have been expected. More research is needed to better understand whether and how attention and expectancies play an interactive role in contagious itch.

It is not yet known whether the underlying neurobiological mechanisms of contagious itch and nocebo effects in cutaneously evoked itch are similar. Conceptually, the experience of contagious itch has been linked to nocebo effects before.^16^, ^37^, ^38^ In a sense, this comparison seems reasonable: contagious itch can be elicited by stimuli from the surrounding context (i.e., itch‐related cues). Experimental studies also show that initially neutral contextual cues can become associated with itch and may exacerbate physical itch sensations (i.e., conditioned nocebo effects in itch, for instance, using abstract cues such as colours^39^, ^40^). However, nocebo effects have always been studied explicitly with physical or chemical induction of itch (i.e., cutaneous itch), which is different from contagious itch elicited by non‐physical cues such as sounds or pictures. The neurobiological underpinnings of nocebo effects in itch, as well as those of contagious itch, are not yet well understood. Current evidence points towards a role of brain areas involved in the somatosensory processing of contagious itch.^1^, ^41^, ^42^, ^43^ It has also been hypothesized that activation of the mirror neuron system may be relevant.^4^, ^44^ There is some data supporting this: among other, brain regions involved in the simulation of actions also appear involved in contagious itch.^1^, ^45^ These areas differ from the brain areas that have been associated with nocebo effects following cutaneous itch induction (e.g., the caudate, and dorsolateral prefrontal cortex): those are generally involved in other functions, such as cognitive executive and motivational processing.^41^ Differing neural circuits may potentially explain why contagious itch would be less sensitive to change following suggestions, but this needs to be confirmed in future research. Moreover, no study to date has investigated the neurobiological mechanisms of placebo effects in itch. If these neural underpinnings are found to be different between placebo and nocebo effects and contagious itch, it may confirm that they are two distinct phenomena. Particularly, contagious itch may be an evolutionary ingrained defence system^46^; if so, this could mean that it may not be particularly sensitive to expectancy effects.

In the current study, higher self‐assessed sensitive skin was associated with larger differences in contagious itch intensity between scratching and rubbing sounds in healthy, itch‐free individuals. These findings complement prior findings that itch and scratching contagion are elevated in patients with skin disorders e.g.,^3^, ^5^, ^43^, ^47^ For instance, prior research demonstrates that itch in response to scratching sounds is amplified in psoriatic patients compared with healthy individuals.^5^ This is the first time that an association between more sensitive skin and higher auditory itch contagion following scratching sounds has been found outside of a clinical population. Sensitive skin is associated with more frequent and intense subjective complaints, such as itch, in response to external stimuli.^48^ Individuals with more sensitive skin may be more likely to experience itch and may, as a consequence, be exposed more frequently to scratching sounds. This could in theory have sensitized them to contagious itch. More research is needed to replicate these findings.

Some limitations of the current work need to be addressed. The study was conducted online because of the global COVID‐19 pandemic. As participants could have listened to the scratching and rubbing sounds from any device, differences in sound quality (e.g., the speaker system, in‐ear headphones, noise‐cancelling headphones) may have influenced the findings. Participants were also able to control the volume of the sounds themselves. It cannot be ruled out that participants who found the sounds to be unpleasant may have listened to them at a lower volume compared with other participants, which could have altered itch experience. In addition, the suggestions consisted of an approximately half a page of text, which participants may or may not have read thoroughly. Repetition of the study in a controlled laboratory environment is advisable to control for these external factors. Moreover, there was no baseline measurement of contagious itch. As such, it is unclear how the participants in the suggestion groups would have experienced contagious itch if no suggestions were given. Future research may consider adding such a baseline assessment, for instance, by investigating itch in two separate (counterbalanced) control and verbal suggestions sessions. Finally, we recommended that future research embeds the itch assessments in a broader array of sensations (e.g., bothersome, painful, unpleasant), to avoid induction of itch expectancies. This may improve itch assessments within a control group in particular.

In conclusion, the current study replicates previous findings on auditory itch contagion that show that scratching sounds elicit more itch than rubbing sounds, and that itch intensity increases as a function of high‐frequency amplitude. The difference in itch intensity following scratching and rubbing sounds was absent in the positive and negative suggestions groups, where participants experienced itch of equal magnitude in response to both types of auditory stimuli. These findings show that, though suggestions can modulate contagious itch to some degree, their effect on contagious itch as a whole is not straightforward. More research on the role of expectancies in contagious itch is needed. Moreover, the findings highlight that care may be needed in communicating about somatic symptoms such as itch.

## AUTHOR CONTRIBUTIONS

All authors contributed to the design of the study. Stefanie H. Meeuwis and Aleksandrina Skvortsova performed the research, conducted the statistical analyses, and wrote the first draft of the manuscript. Andrea W. M. Evers, Henning Holle, Antoinette I. M. van Laarhoven provided input for the manuscript and commented on the text. All authors read and approved of the final draft.

## FUNDING INFORMATION

This work was supported by a NWO VICI Grant (number: 45316004) granted to AE.

## CONFLICT OF INTEREST

The authors declare no conflict of interest.

## Supporting information


**APPENDIX 1** Instructions provided prior to the scratching and rubbing sounds
**APPENDIX 2** Secondary analysis of replicability of prior research findings across groups
**FIGURE S1** Mean itch ratings ± standard error, plotted across sound type (scratching and rubbing sounds) and across high frequency (HF) tones’ amplitude (−10 decibel, original recording, +10 decibel)
**FIGURE S2** Individual data points and box plots of itch scores by sound type (scratching, rubbing) and by HF amplitude, plotted separately for the positive suggestions (*n* = 51), negative suggestions (*n* = 41), and control group (*n* = 41)
**APPENDIX 3** Moderation of group effects by interindividual differences
**FIGURE S3** The difference in itch elicited by scratching compared to rubbing sounds changed significantly across sensitive skin (SS10) ratings for the control group, but not for the negative suggestions group (see Table S3 for the statistical data). Moderation analysis indicates that the difference in itch between scratching and rubbing sounds was significant for medium (M) and high (+1 SD), but not for low (−1 SD) levels of sensitive skin in the control group. The difference was non‐significant for the negative suggestions group regardless of sensitive skin ratings
**FIGURE S4** Itch levels evoked by the scratching sounds (A) and rubbing sounds (B) respectively, within the negative suggestions group and control group and plotted across low (−1 SD), medium (M) and high (+1 SD) levels of sensitive skin (SS10). Even though no significant group × SS10 interaction effect was found for itch elicited by either scratching or rubbing sounds, differences in how itch changes across levels of sensitive skin for each group may have contributed to the significant group × SS10 × movement type interaction (see also Figure S3 and Table S3)
**FIGURE S5** The difference in auditory itch elicited by scratching compared to rubbing sounds changed significantly across sensitive skin (SS10) ratings for the control group, but not for the positive suggestions group (see Table S2 for the statistical data). Moderation analysis indicates that the difference in auditory itch between scratching and rubbing sounds was significant for medium (M) and high (+1 SD), but not for low (−1 SD) levels of sensitive skin in the control group. The difference was non‐significant for the positive suggestions group regardless of sensitive skin ratings
**FIGURE S6** Itch levels evoked by the scratching sounds (**A**) and rubbing sounds (**B)** respectively, within the positive suggestions group and control group and plotted across low (−1 SD), medium (M) and high (+1 SD) levels of sensitive skin (SS10). Even though no significant group × SS10 interaction effect was found for itch elicited by either scratching or rubbing sounds, differences in how itch changes across levels of sensitive skin for each group may have contributed to the significant group × SS10 × movement type interaction (see also Figure S5 and Table S2)

**TABLE S1** Pearson's correlation coefficients for the associations between interindividual differences and itch elicited by the scratching and rubbing sounds across all groups
**TABLE S2** Summary of the tests for moderation of the effects of group by interindividual differences, with itch elicited by scratching and rubbing sounds in the positive suggestions group (*n* = 52) versus the control instructions group (*n* = 42) as outcome
**TABLE S3** Summary of the tests for moderation of the effects of group by interindividual differences, with itch elicited by scratching and rubbing sounds in the negative suggestions group (*n* = 46) versus the control instructions group (*n* = 42) as outcome

## Data Availability

The data that support the findings of this study are openly available in OSF at https://osf.io/muqcj/, reference number 10.17605/OSF.IO/MUQCJ.
