# Supplementary material for: Can contagious itch be affected by positive and negative suggestions?
Source: Exp Dermatol. 2022 Sep 1;31(12):1853–62. doi: 10.1111/exd.14663 (PMC10087404; doi:10.1111/exd.14663)
Supplement: Supplementary file 1 — APPENDIX 1 Instructions provided prior to the scratching and rubbing sounds APPENDIX 2 Secondary analysis of replicability of prior research findings across groups FIGURE S1 Mean itch ratings ± standard error, plotted across sound type (scratching and rubbing sounds) and across high frequency (HF) tones’ amplitude (−10 decibel, original recording, +10 decibel) FIGURE S2 Individual data points and box plots of itch scores by sound type (scratching, rubbing) and by HF amplitude, plotted separately for the positive suggestions (n = 51), negative suggestions (n = 41), and control group (n = 41) APPENDIX 3 Moderation of group effects by interindividual differences FIGURE S3 The difference in itch elicited by scratching compared to rubbing sounds changed significantly across sensitive skin (SS10) ratings for the control group, but not for the negative suggestions group (see Table S3 for the statistical data). Moderation analysis indicates that the difference in itch between scratching and rubbing sounds was significant for medium (M) and high (+1 SD), but not for low (−1 SD) levels of sensitive skin in the control group. The difference was non‐significant for the negative suggestions group regardless of sensitive skin ratings FIGURE S4 Itch levels evoked by the scratching sounds (A) and rubbing sounds (B) respectively, within the negative suggestions group and control group and plotted across low (−1 SD), medium (M) and high (+1 SD) levels of sensitive skin (SS10). Even though no significant group × SS10 interaction effect was found for itch elicited by either scratching or rubbing sounds, differences in how itch changes across levels of sensitive skin for each group may have contributed to the significant group × SS10 × movement type interaction (see also Figure S3 and Table S3) FIGURE S5 The difference in auditory itch elicited by scratching compared to rubbing sounds changed significantly across sensitive skin (SS10) ratings for the control group, but not for the posit [file EXD-31-1853-s002.zip › EXD_14663_Appendix 1.docx]

**Appendix 1.**

**Instructions provided prior to the scratching and rubbing sounds**

***Negative suggestions group***.

In the next part of this study, you will listen to various sound fragments. It will be scratching and rubbing sounds. Previous research has shown that itch is a highly socially contagious sensation: listening to a lecture about itch or listening to scratching sounds can elicit an intense feeling of itch and desire to scratch in most people. The sounds you will listen to have been found to elicit sensations of itch as well.

Contagious itch originates from activation of mirror neurons in the itch-scratch matrix of the brain. Mirror neurons activate when seeing behaviour in others and stimulate the observer to copy the behaviours. This activation is especially strong in case of scratching: sounds associated with the behaviour (i.e., scratching sounds) already result in a desire to scratch and intense itching sensations. The strong response to scratching sounds is likely evolutionary, and stems from the need to avoid itch-related diseases, bacteria and insects such as lice.

Itch is also contagious when people are aware of this phenomenon. In fact, research has demonstrated that being aware of the contagiousness of itch can even increase its impact significantly.

In this study we want to replicate the previous research, and see whether being aware of contagiousness of itch will amplify your itch sensation triggered by sounds of rubbing and scratching.

Please listen to the sounds carefully. You will be asked afterwards how much itch you experienced while listening to the sound fragment. Please do not think too much about what to respond but rather give the first answer that comes to mind.

***Positive suggestions group****.*

In the next part of this study, you will listen to various sound fragments. It will be scratching and rubbing sounds. Previous research has shown that itch is a possibly socially contagious sensation, unless a person knows that itch is contagious. Thus, listening to a lecture about itch or listening to scratching sounds can elicit a feeling of itch and desire to scratch in most people, but this is no longer true when you are aware about the fact that itch can be contagious.

Contagious itch originates from activation of mirror neurons in the itch-scratch matrix of the brain which is an unconscious process. Mirror neurons activate when seeing behaviour in others and stimulate the observer to copy the behaviours. In case of itch, scratching sounds are associated with scratching which can lead to the sensation of itch. Research has demonstrated, however, that being aware of the contagiousness of itch can significantly reduce its impact. As soon as a person knows this, mirror neurons do not activate anymore. It is still largely unknown why being aware of this phenomenon blocks the activation of mirror neurons. However, it is hypothesized that conscious expectations lead to stronger brain signals, that prevent mirror neurons from activating.

In this study we want to replicate the previous research, and see whether being aware of contagiousness of itch will block this sensation.

Please listen to the sounds carefully. You will be asked afterwards how much itch you experienced while listening to the sound fragment. Please do not think too much about what to respond but rather give the first answer that comes to mind.

***Control group.***

In the next part of this study, you will listen to various sound fragments.

With this study we would like to investigate the individual differences in the sensitivity to itch in response to various sounds, and the role that psychological factors may have in explaining these differences. Using the knowledge obtained in this study, we hope to get a better understanding of the underlying mechanisms of itch sensitivity, and the impact that psychological factors have on it.

The fragments you will listen to all contain scratching and rubbing sounds.

Please listen to the sounds carefully. You will be asked afterwards how much itch you experienced while listening to the sound fragment. Please do not think too much about what to respond but rather give the first answer that comes to mind.
