# Supplementary material for: Can contagious itch be affected by positive and negative suggestions?
Source: Exp Dermatol. 2022 Sep 1;31(12):1853–62. doi: 10.1111/exd.14663 (PMC10087404; doi:10.1111/exd.14663)
Supplement: Supplementary file 1 — APPENDIX 1 Instructions provided prior to the scratching and rubbing sounds APPENDIX 2 Secondary analysis of replicability of prior research findings across groups FIGURE S1 Mean itch ratings ± standard error, plotted across sound type (scratching and rubbing sounds) and across high frequency (HF) tones’ amplitude (−10 decibel, original recording, +10 decibel) FIGURE S2 Individual data points and box plots of itch scores by sound type (scratching, rubbing) and by HF amplitude, plotted separately for the positive suggestions (n = 51), negative suggestions (n = 41), and control group (n = 41) APPENDIX 3 Moderation of group effects by interindividual differences FIGURE S3 The difference in itch elicited by scratching compared to rubbing sounds changed significantly across sensitive skin (SS10) ratings for the control group, but not for the negative suggestions group (see Table S3 for the statistical data). Moderation analysis indicates that the difference in itch between scratching and rubbing sounds was significant for medium (M) and high (+1 SD), but not for low (−1 SD) levels of sensitive skin in the control group. The difference was non‐significant for the negative suggestions group regardless of sensitive skin ratings FIGURE S4 Itch levels evoked by the scratching sounds (A) and rubbing sounds (B) respectively, within the negative suggestions group and control group and plotted across low (−1 SD), medium (M) and high (+1 SD) levels of sensitive skin (SS10). Even though no significant group × SS10 interaction effect was found for itch elicited by either scratching or rubbing sounds, differences in how itch changes across levels of sensitive skin for each group may have contributed to the significant group × SS10 × movement type interaction (see also Figure S3 and Table S3) FIGURE S5 The difference in auditory itch elicited by scratching compared to rubbing sounds changed significantly across sensitive skin (SS10) ratings for the control group, but not for the posit [file EXD-31-1853-s002.zip › EXD_14663_Appendix 2_clean.docx]

**Appendix 2.**

**Secondary analysis of replicability of prior research findings across groups**

Methods

Prior research shows that the intensity of itch induced by itch-associated sounds is modulated by the type of sound participant listen to: scratching sounds elicit more itch relative to rubbing sounds. Moreover, the amplitude of the high frequency (HF) tones contained within the sound are associated with itch intensity (with higher HF amplitude eliciting more itch; Swithenbank et al., 2016). These findings were replicated in the control group of the current study (see the results section of the main paper).We examined these patterns in an explorative 3 x 2 x 3 mixed analysis of variance (ANOVA) with group (negative suggestions, positive suggestions, control) as a between-subjects factor, and sound type (scratching, rubbing) and HF amplitude (-10 dB, original, +10 dB) as within-subjects factors. As Mauchly’s test of sphericity indicated that this assumption was violated for HF amplitude (χ^2^ (2) = 18.5, *p* < .001) and for sound type x HF amplitude interaction (χ^2^ (2) =9.7, *p* = .008), Huynh-Feldt corrections were applied. Prior to the analysis, itch scores were square-root transformed (sqrt). The results of the analysis are described below. All values are reported as arithmetic means ± SD unless stated otherwise.

Results

The 3 x 2 x 3 mixed ANOVA demonstrated no significant main effect of group on itch_sqrt_ [F(2,130) = 0.17, *p* = .84, η^2^_partial_ < .01]. A significant main effect was observed for sound type [scratching vs. rubbing; F(1,130) = 22.08, *p* < .001, η^2^_partial_ = .15]. In general, participants rated itch for scratching sounds as more intense (2.35 ± 2.10) relative to rubbing sounds (2.10 ± 1.91; see **Figure S1 & S2** below). A significant interaction between sound type and HF amplitude was found [F(1.92, 249.60) = 4.64, *p* = .011, η^2^_partial_ = .03]. Post-hoc tests showed that the increase in itch depending on HF amplitude was steeper and significant only for the rubbing sounds (pairwise comparisons: *p* _-10dB – original_ = .029, *p* _-10dB – +10dB_ < .001; **Figure S1**), but not for the scratching sounds (both *p* ≥ .15).

A significant interaction between group and sound type moreover demonstrated that the differences in itch between the scratching and rubbing sounds can be mainly found within the control group exclusively [F(2,130) = 5.98, *p* = .003, η^2^_partial_ = .08]. Indeed, post-hoc pairwise comparisons revealed that, in the control group, scratching sounds (2.52 ± 2.41) were rated as more itchy relative to rubbing sounds (2.00 ± 2.10; *p* < .001). By contrast, in both of the suggestion groups, itch ratings for scratching and rubbing sounds did not differ (both *p* ≥ .16). When groups were compared post-hoc for each sound type individually, no differences in itch were observed (both *p* ≥ .86).

The mixed ANOVA also revealed a significant main effect of HF amplitude on itch_sqrt_ [F(1.81, 235.87) = 12.49, *p* < .001, η^2^_partial_ = .09]. Post-hoc pairwise comparisons indicated that for every +10 dB increase in HF amplitude, itch intensity increased as well (both *p* ≤ .041; **Figure S1**). A significant interaction between group and HF amplitude again demonstrated that this finding can be mainly attributed to the differences in itch within the control group [F(3.63, 235.87) = 3.06, *p* = .021, η^2^_partial_ = .05]. Within the control group, itch was higher for high HF amplitude (+10 dB; 2.73 ± 2.56) relative to when HF amplitude was lower (i.e., post-hoc comparisons: original HF amplitude: 2.50 ± 2.47, *p* = .016; -10 dB: 2.35 ± 2.34, *p* < .001). Within the suggestion groups, itch did not differ regardless of HF amplitude (all *p* ≥ .28). Comparisons between groups on each HF amplitude level individually revealed no significant differences in itch (all *p* ≥ .96).

Taken together, the findings show that the increases in itch for each increase in HF amplitude were steeper for the rubbing sounds relative to the scratching sounds. Moreover, any difference in itch between scratching sounds and rubbing sounds, and between different levels of HF amplitude can be found within the control group. In both suggestion groups, participants rated itch equally regardless of sound type or HF amplitude.

**References**

Swithenbank, S., Cowdell, F., & Holle, H. (2016). The role of auditory itch contagion in psoriasis. Acta dermato-venereologica, 96(6), 728-736.


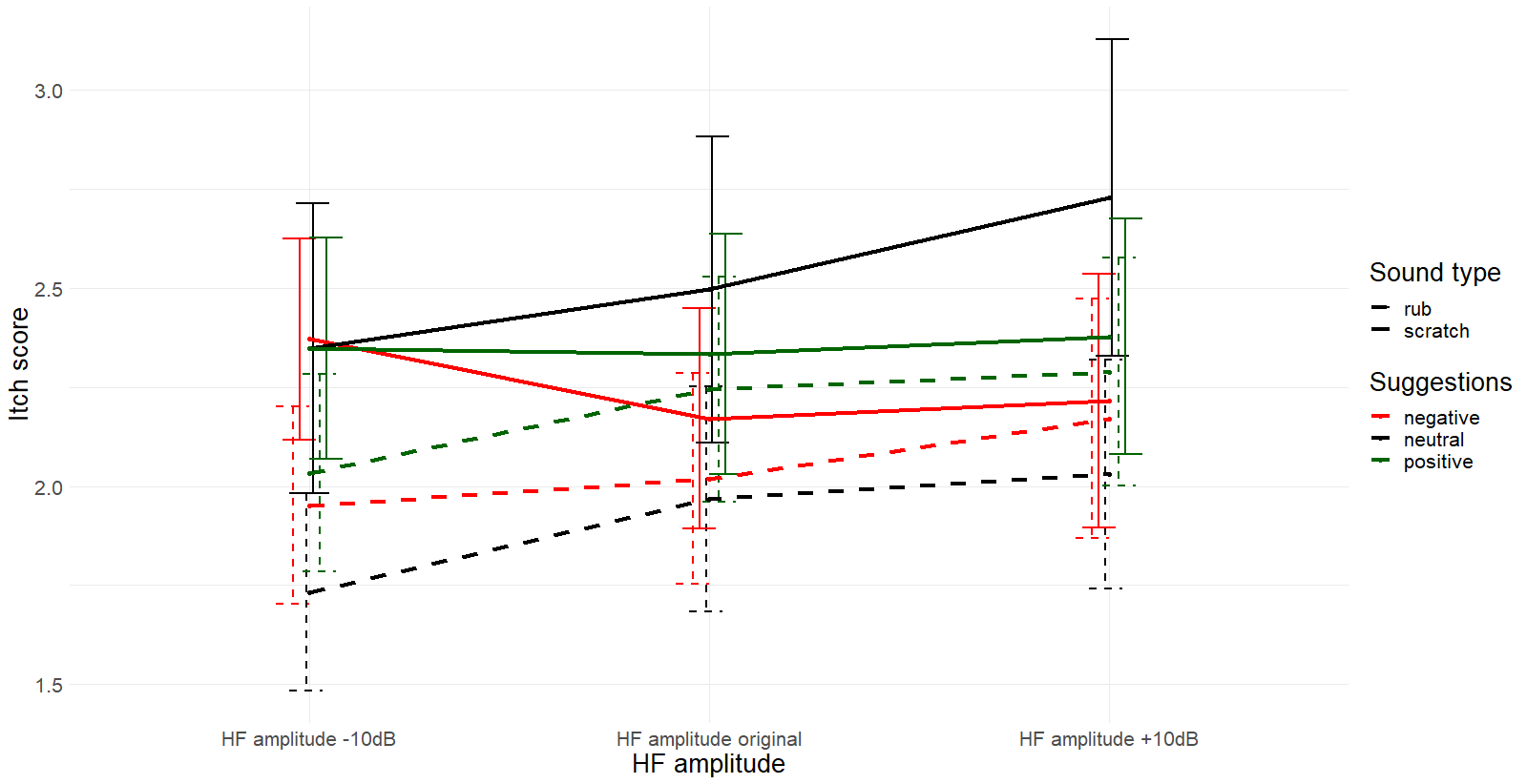


**Figure S1.** Mean itch ratings ± standard error, plotted across sound type (scratching and rubbing sounds) and across high frequency (HF) tones’ amplitude (-10 decibel, original recording, +10 decibel).


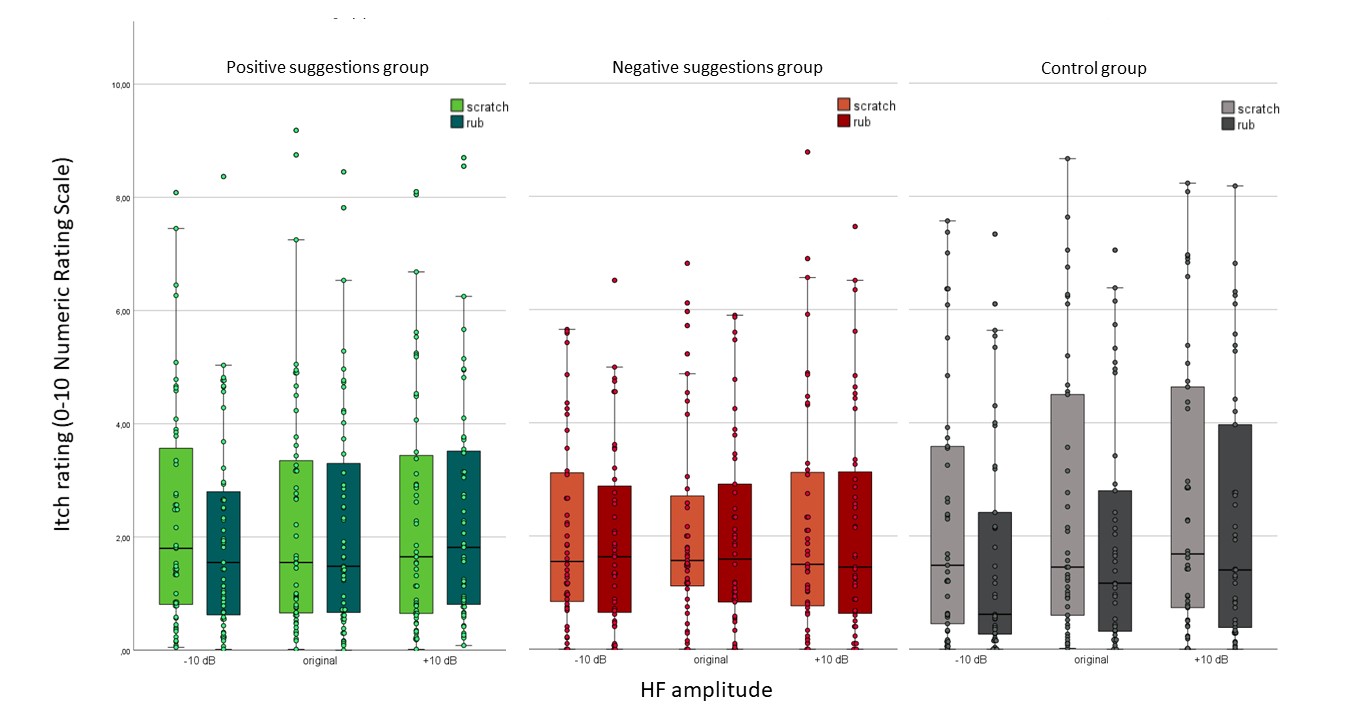
**Figure S2**. Individual data points and box plots of itch scores by sound type (scratching, rubbing) and by HF amplitude, plotted separately for the positive suggestions (n = 51), negative suggestions (n = 41), and control group (n = 41).
