# Supplementary material for: Can contagious itch be affected by positive and negative suggestions?
Source: Exp Dermatol. 2022 Sep 1;31(12):1853–62. doi: 10.1111/exd.14663 (PMC10087404; doi:10.1111/exd.14663)
Supplement: Supplementary file 1 — APPENDIX 1 Instructions provided prior to the scratching and rubbing sounds APPENDIX 2 Secondary analysis of replicability of prior research findings across groups FIGURE S1 Mean itch ratings ± standard error, plotted across sound type (scratching and rubbing sounds) and across high frequency (HF) tones’ amplitude (−10 decibel, original recording, +10 decibel) FIGURE S2 Individual data points and box plots of itch scores by sound type (scratching, rubbing) and by HF amplitude, plotted separately for the positive suggestions (n = 51), negative suggestions (n = 41), and control group (n = 41) APPENDIX 3 Moderation of group effects by interindividual differences FIGURE S3 The difference in itch elicited by scratching compared to rubbing sounds changed significantly across sensitive skin (SS10) ratings for the control group, but not for the negative suggestions group (see Table S3 for the statistical data). Moderation analysis indicates that the difference in itch between scratching and rubbing sounds was significant for medium (M) and high (+1 SD), but not for low (−1 SD) levels of sensitive skin in the control group. The difference was non‐significant for the negative suggestions group regardless of sensitive skin ratings FIGURE S4 Itch levels evoked by the scratching sounds (A) and rubbing sounds (B) respectively, within the negative suggestions group and control group and plotted across low (−1 SD), medium (M) and high (+1 SD) levels of sensitive skin (SS10). Even though no significant group × SS10 interaction effect was found for itch elicited by either scratching or rubbing sounds, differences in how itch changes across levels of sensitive skin for each group may have contributed to the significant group × SS10 × movement type interaction (see also Figure S3 and Table S3) FIGURE S5 The difference in auditory itch elicited by scratching compared to rubbing sounds changed significantly across sensitive skin (SS10) ratings for the control group, but not for the posit [file EXD-31-1853-s002.zip › EXD_14663_Appendix 3_clean.docx]

## Appendix 3.

**Moderation of group effects by interindividual differences**

l (n=42)

1.20

**Group**

Contro Negati

1.00

0.80

0.60

0.40

0.20

0.00

**Difference score for itch (scratching - rubbing)**

ve (n=46)

0.00

5.00

10.00

15.00

20.00

25.00

30.00

## Sensitive skin (SS10)

**Supplementary Figure S3.** The difference in itch elicited by scratching compared to rubbing sounds changed significantly across sensitive skin (SS10) ratings for the control group, but not for the negative suggestions group (see Table 4 for the statistical data). Moderation analysis indicates that the difference in itch between scratching and rubbing sounds was significant for medium (M) and high (+1 SD), but not for low (-1 SD) levels of sensitive skin in the control group. The difference was non-significant for the negative suggestions group regardless of sensitive skin ratings.

# [A]

3.50

3.00

**Itch following scratching sounds**

2.50

2.00

1.50

## Group

*Appendix 3*

0.00

Control (n=42) Negative (n=46)

5.00

10.00

15.00

20.00

25.00

30.00

# [B]

3.50

3.00

**Itch following rubbing sounds**

2.50

2.00

1.50

## Sensitive skin (SS10)

0.00

5.00

10.00

15.00

20.00

25.00

30.00

## Sensitive skin (SS10)

**Supplementary Figure S4.** Itch levels evoked by the scratching sounds [**A**] and rubbing sounds [**B**] respectively, within the negative suggestions group and control group and plotted across low (-1 SD), medium (M) and high (+1 SD) levels of sensitive skin (SS10). Even though no significant group x SS10 interaction effect was found for itch elicited by either scratching or rubbing sounds, differences in how itch changes across levels of sensitive skin for each group may have contributed to the significant group x SS10 x movement type interaction (see also Supplementary Figure S1 & Table 4).

*Appendix 3*

l (n=42) e (n=52)

1.20

**Group**

Contro Positiv

1.00

0.80

0.60

0.40

0.20

0.00

**Difference score for auditory itch (scratching - rubbing)**

0.00

5.00

10.00

15.00

20.00

25.00

30.00

## Sensitive skin (SS10)

**Supplementary Figure S5.** The difference in auditory itch elicited by scratching compared to rubbing sounds changed significantly across sensitive skin (SS10) ratings for the control group, but not for the positive suggestions group (see Table 5 for the statistical data). Moderation analysis indicates that the difference in auditory itch between scratching and rubbing sounds was significant for medium (M) and high (+1 SD), but not for low (-1 SD) levels of sensitive skin in the control group. The difference was non-significant for the positive suggestions group regardless of sensitive skin ratings.

*Appendix 3*

# [A]

3.50

3.00

**Itch following scratching sounds**

2.50

2.00

1.50

## Group

0.00

Control (n=42) Positive (n=52)

5.00

10.00

15.00

20.00

25.00

30.00

# [B]

3.50

3.00

**Itch following rubbing sounds**

2.50

2.00

1.50

## Sensitive skin (SS10)

0.00

5.00

10.00

15.00

20.00

25.00

30.00

## Sensitive skin (SS10)

**Supplementary Figure S6**. Itch levels evoked by the scratching sounds [**A**] and rubbing sounds [**B]** respectively, within the positive suggestions group and control group and plotted across low (-1 SD), medium (M) and high (+1 SD) levels of sensitive skin (SS10). Even though no significant group x SS10 interaction effect was found for itch elicited by either scratching or rubbing sounds, differences in how itch changes across levels of sensitive skin for each group may have contributed to the significant group x SS10 x movement type interaction (see also Supplementary Figure S3 & Table 5).
