# Supplementary material for: Can contagious itch be affected by positive and negative suggestions?
Source: Exp Dermatol. 2022 Sep 1;31(12):1853–62. doi: 10.1111/exd.14663 (PMC10087404; doi:10.1111/exd.14663)
Supplement: Supplementary file 2 — TABLE S1 Pearson's correlation coefficients for the associations between interindividual differences and itch elicited by the scratching and rubbing sounds across all groups TABLE S2 Summary of the tests for moderation of the effects of group by interindividual differences, with itch elicited by scratching and rubbing sounds in the positive suggestions group (n = 52) versus the control instructions group (n = 42) as outcome TABLE S3 Summary of the tests for moderation of the effects of group by interindividual differences, with itch elicited by scratching and rubbing sounds in the negative suggestions group (n = 46) versus the control instructions group (n = 42) as outcome [file EXD-31-1853-s001.docx]

**Supplementary materials**

**Supplementary Table S1**. Pearson’s correlation coefficients for the associations between interindividual differences and itch elicited by the scratching and rubbing sounds across all groups.

|  | Itch | |
| --- | --- | --- |
|  | Scratching sounds | Rubbing sounds |
| Neuroticism | -.001 | -.03 |
| Extraversion | -.05 | -.02 |
| Optimism | -.14 | -.10 |
| Worrying | .01 | -.01 |
| State anxiety | .13 | .16 † |
| Sensitive skin ratings | .22 * | .19 * |

Note. *** *p* < .001, ** *p* < .01, * *p* < .05, † *p* < .10. n = 6 is missing. Itch following scratching and rubbing sounds was averaged across HF amplitude. Correlation coefficients were calculated using square-root transformed itch scores.

**Supplementary Table S2**. Summary of the tests for moderation of the effects of group by interindividual differences, with itch elicited by scratching and rubbing sounds in the positive suggestions group (n = 52) versus the control instructions group (n = 42) as outcome.

|  | | Neuroticism | | Extraversion | | Optimism | | Worrying | | State Anxiety | | Sensitive skin | |
| --- | --- | --- | --- | --- | --- | --- | --- | --- | --- | --- | --- | --- | --- |
|  | | b (SE) | 95% CI | b (SE) | 95% CI | b (SE) | 95% CI | b (SE) | 95% CI | b (SE) | 95% CI | b (SE) | 95% CI |
|  | |  | | | |  |  |  |  |  |  |  |  |
|  | | **Model 1: slope (scratching - rubbing difference)** | | | |  |  |  |  |  |  |  |  |
| Group | | -0.03 (0.07) | [-0.18, 0.12] | <0.01 (0.09) | [-0.17, 0.18] | -0.11 (0.13) | [-0.37, 0.15] | -0.05 (0.16) | [-0.37, 0.27] | -0.21 (0.13) | [-0.47, 0.05] | 0.06 (0.06) | [-0.06, 0.17] |
| Interindividual difference | | 0.01 (0.01) | [-0.004, 0.03] | 0.01 (0.01) | [-0.01, 0.02] | >-0.01 (0.01) | [-0.02, 0.01] | <0.01 (<0.01) | [>-0.01, 0.01] | -0.01 (0.01) | [-0.03, 0.01] | 0.01 (<0.01)*** | [0.01, 0.02] |
| Group x Int. diff. | | -0.01 (0.01) | [-0.04, 0.01] | -0.01 (0.01) | [-0.03, 0.01] | <0.01 (0.01) | [-0.02, 0.02] | >-0.01 (<0.01) | [-0.01, 0.01] | 0.01 (0.01) | [-0.01, 0.03] | -0.01 (<0.01)*** | [-0.02, >-0,01] |
|  | |  |  |  |  |  |  |  |  |  |  |  |  |
| F(model) | | 3.24* |  | 3.18* |  | 2.79* |  | 2.56† |  | 2.80* |  | 9.17*** |  |
| R^2^ | | .10 |  | .10 |  | .09 |  | .08 |  | .09 |  | .24 |  |
|  | |  |  |  |  |  |  |  |  |  |  |  |  |
| Conditional effects at moderator level: | Control, -1SD | - | - | - | - | - | - | - | - | - | - | 0.02 (0.04) | [-0.05, 0.10] |
|  | Control, M | - | - | - | - | - | - | - | - | - | - | 0.14 (0.03)*** | [0.08, 0.19] |
|  | Control, +1SD | - | - | - | - | - | - | - | - | - | - | 0.25 (0.04)*** | [0.18, 0.32] |
|  | Sugg., -1SD | - | - | - | - | - | - | - | - | - | - | 0.04 (0.03) | [-0.02, 0.11] |
|  | Sugg., M | - | - | - | - | - | - | - | - | - | - | 0.03 (0.02) | [-0.02, 0.08] |
|  | Sugg., +1SD | - | - | - | - | - | - | - | - | - | - | 0.02 (0.03) | [-0.04, 0.09] |
|  | |  |  |  |  |  |  |  |  |  |  |  |  |
|  | | **Model 2: itch for scratching sounds** | | | |  |  |  |  |  |  |  |  |
| Group | | 0.09 (0.24) | [-0.38, 0.56] | 0.35 (0.28) | [-0.21, 0.91] | 0.04 (0.41) | [-0.78, 0.87] | 0.29 (0.51) | [-0.72, 1.30] | 0.20 (0.41) | [-0.62, 1.01] | -0.05 (0.19) | [-0.43, 0.34] |
| Interindividual difference | | <0.01 (0.03) | [-0.05, 0.05] | 0.02 (0.03) | [-0.03, 0.07] | -0.01 (0.02) | [-0.05, 0.03] | <0.01 (0.01) | [-0.01, 0.02] | 0.03 (0.03) | [-0.02, 0.09] | 0.01 (,0.01) | [>-0.01, 0.03] |
| Group x Int. diff. | | -0.02 (0.04) | [-0.09, 0.05] | -0.05 (0.03) | [-0.11, 0.02] | >-0.01 (0.03) | [-0.06, 0.05] | -0.01 (0.01) | [-0.02, 0.01] | -0.02 (0.03) | [-0.09, 0.05] | <0.01 (0.01) | [-0.02, 0.02] |
|  | |  |  |  |  |  |  |  |  |  |  |  |  |
| F(model) | | 0.20 |  | 0.79 |  | 0.29 |  | 0.15 |  | 0.63 |  | 1.99 |  |
| R^2^ | | <.01 |  | .03 |  | .01 |  | 0.01 |  | .02 |  | .06 |  |
|  | |  |  |  |  |  |  |  |  |  |  |  |  |
|  | | **Model 2: itch for rubbing sounds** | | | |  |  |  |  |  |  |  |  |
| Group | | 0.12 (0.22) | [-0.31, 0.56] | 0.35 (0.26) | [-0.18, 0.87] | 0.15 (0.39) | [-0.62, 0.92] | 0.34 (0.47) | [-0.59, 1.28] | 0.40 (0.38) | [-0.35, 1.16] | -0.11 (0.18) | [-0.46, 0.25] |
| Interindividual difference | | -0.01 (0.02) | [-0.06, 0.04] | 0.02 (0.02) | [-0.03, 0.06] | -0.01 (0.02) | [-0.05, 0.03] | <0.01 (0.01) | [-0.01, 0.02] | 0.04 (0.03) | [-0.01, 0.09] | <0.01 (0.01) | [-0.01, 0.02] |
| Group x Int. diff. | | -0.01 (0.03) | [-0.07, 0.06] | -0.03 (0.03) | [-0.09, 0.03] | >-0.01 (0.03) | [-0.06, 0.05] | >-0.01 (0.01) | [-0.02, 0.01] | -0.03 (0.03) | [-0.09, 0.03] | 0.01 (0.01) | [-0.01, 0.03] |
|  | |  |  |  |  |  |  |  |  |  |  |  |  |
| F(model) | | 0.45 |  | 0.65 |  | 0.35 |  | 0.30 |  | 1.19 |  | 1.87 |  |
| R^2^ | | .02 |  | .02 |  | .01 |  | .01 |  | .04 |  | .06 |  |
|  | |  |  |  |  |  |  |  |  |  |  |  |  |

Note. *** *p* < .001, ** *p* < .01, * *p* < .05, † *p* < .10. Int. diff. = interindividual difference; Sugg. = suggestions. Itch following scratching and rubbing sounds was averaged across HF amplitude. The analyses were conducted using square-root transformed itch scores.

**Supplementary Table S3**. Summary of the tests for moderation of the effects of group by interindividual differences, with itch elicited by scratching and rubbing sounds in the negative suggestions group (n = 46) versus the control instructions group (n = 42) as outcome.

|  | | Neuroticism | | Extraversion | | Optimism | | Worrying | | State Anxiety | | Sensitive skin | |
| --- | --- | --- | --- | --- | --- | --- | --- | --- | --- | --- | --- | --- | --- |
|  | | b (SE) | 95% CI | b (SE) | 95% CI | b (SE) | 95% CI | b (SE) | 95% CI | b (SE) | 95% CI | b (SE) | 95% CI |
|  | |  |  |  |  |  |  |  |  |  |  |  |  |
|  | | **Model 1: slope (scratching - rubbing difference)** | | | |  |  |  |  |  |  |  |  |
| Group | | -0.03 (0.09) | [-0.21, 0.14] | 0.05 (0.10) | [-0.16, 0.26] | -0.10 (0.15) | [-0.40, 0.21] | -0.10 (0.19) | [-0.48, 0.27] | -0.21 (0.15) | [-0.52, 0.10] | 0.07 (0.06) | [-0.06, 0.19] |
| Interindividual difference | | 0.01 (0.01) | [-0.01, 0.03] | 0.01 (0.01) | [-0.01, 0.02] | >-0.01 (0.01) | [-0.02, 0.01] | <0.01 (<0.01) | [>-0.01, 0.01] | -0.01 (0.01) | [-0.03, 0.01] | 0.01 (<0.01)*** | [0.01, 0.02] |
| Group x Int. diff. | | -0.01 (0.01) | [-0.04, 0.01] | -0.02 (0.01) | [-0.05, <0.01] | >-0.01 (0.01) | [-0.02, 0.02] | >-0.01 (<0.01) | [-0.01, 0.01] | 0.01 (0.01) | [-0.02, 0.04] | -0.01 (<0.01)*** | [-0.02, -0.01] |
|  | |  |  |  |  |  |  |  |  |  |  |  |  |
| F(model) | | 2.68† |  | 3.10* |  | 2.31† |  | 2.14 |  | 2.30† |  | 8.05*** |  |
| R^2^ | | .09 |  | .11 |  | .08 |  | .08 |  | .08 |  | .24 |  |
|  | |  |  |  |  |  |  |  |  |  |  |  |  |
| Conditional effects at moderator level: | Control, -1SD | - | - | - | - | - | - | - | - | - | - | 0.02 (0.04) | [-0.06, 0.10] |
|  | Control, M | - | - | - | - | - | - | - | - | - | - | 0.16 (0.03)*** | [0.10, 0.21] |
|  | Control, +1SD | - | - | - | - | - | - | - | - | - | - | 0.29 (0.05)*** | [0.20, 0.39] |
|  | Sugg., -1SD | - | - | - | - | - | - | - | - | - | - | 0.05 (0.04) | [-0.03, 0.13] |
|  | Sugg., M | - | - | - | - | - | - | - | - | - | - | 0.03 (0.03) | [-0.02, 0.09] |
|  | Sugg., +1SD | - | - | - | - | - | - | - | - | - | - | 0.02 (0.03) | [-0.05, 0.09] |
|  | |  |  |  |  |  |  |  |  |  |  |  |  |
|  | | **Model 2: itch for scratching sounds** | | | |  |  |  |  |  |  |  |  |
| Group | | -0.17 (0.25) | [-0.68, 0.33] | 0.18 (0.31) | [-0.43, 0.79] | 0.18 (0.45) | [-0.71, 1.07] | 0.21 (0.55) | [-0.88, 1.30] | 0.02 (0.44) | [-0.87, 0.90] | <0.01 (0.20) | [-0.40, 0.40] |
| Interindividual difference | | <0.01 (0.03) | [-0.05, 0.05] | 0.02 (0.03) | [-0.03, 0.07] | -0.01 (0.02) | [-0.05, 0.03] | <0.01 (0.01) | [-0.01, 0.02] | 0.04 (0.03) | [-0.02, 0.09] | 0.01 (0.01) | [>-0.01, 0.03] |
| Group x Int. diff. | | 0.02 (0.04) | [-0.06, 0.09] | -0.03 (0.04) | [-0.10, 0.04] | -0.02 (0.03) | [-0.08, 0.04] | -0.01 (0.01) | [-0.02, 0.01] | -0.01 (0.04) | [-0.08, 0.06] | -0.01 (0.01) | [-0.03, 0.01] |
|  | |  |  |  |  |  |  |  |  |  |  |  |  |
| F(model) | | 0.24 |  | 0.37 |  | 0.64 |  | 0.19 |  | 0.90 |  | 1.11 |  |
| R^2^ | | .01 |  | .01 |  | .02 |  | .01 |  | .03 |  | .04 |  |
|  | |  |  |  |  |  |  |  |  |  |  |  |  |
|  | | **Model 2: itch for rubbing sounds** | | | |  |  |  |  |  |  |  |  |
| Group | | -0.14 (0.24) | [-0.61, 0.34] | 0.13 (0.29) | [-0.45, 0.71] | 0.28 (0.42) | [-0.57, 1.12] | 0.32 (0.52) | [-0.72, 1,35] | 0.23 (0.42) | [-0.60, 1.05] | -0.06 (0.19) | [-0.45, 0.32] |
| Interindividual difference | | -0.01 (0.02) | [-0.06, 0.04] | 0.02 (0.02) | [-0.03, 0.06] | -0.01 (0.02) | [-0.05, 0.03] | <0.01 (0.01) | [-0.01, 0.02] | 0.04 (0.03) | [-0.01, 0.09] | <0.01 (0.01) | [-0.01, 0.02] |
| Group x Int. diff. | | 0.03 (0.03) | [-0.04, 0.10] | -0.01 (0.03) | [-0.08, 0.06] | -0.02 (0.03) | [-0.07, 0.04] | -0.01 (0.01) | [-0.02, 0.01] | -0.02 (0.04) | [-0.09, 0.05] | 0.01 (0.01) | [-0.01, 0.02] |
|  | |  |  |  |  |  |  |  |  |  |  |  |  |
| F(model) | | 0.34 |  | 0.20 |  | 0.46 |  | 0.16 |  | 1.26 |  | 0.59 |  |
| R^2^ | | .01 |  | .01 |  | .02 |  | .01 |  | .05 |  | .02 |  |
|  | |  |  |  |  |  |  |  |  |  |  |  |  |

Note. *** *p* < .001, ** *p* < .01, * *p* < .05, † *p* < .10. Int. diff. = interindividual difference; Sugg. = suggestions. Itch following scratching and rubbing sounds was averaged across HF amplitude. The analyses were conducted using square-root transformed itch scores.
